# The impact of antifungals on toll-like receptors

**DOI:** 10.3389/fmicb.2014.00099

**Published:** 2014-03-14

**Authors:** Mircea R. Mihu, Rodney Pattabhi, Joshua D. Nosanchuk

**Affiliations:** ^1^Division of Infectious Diseases, Department of Medicine, Albert Einstein College of Medicine–Montefiore Medical CenterBronx, NY, USA; ^2^Department of Microbiology and Immunology, Albert Einstein College of Medicine–Montefiore Medical CenterBronx, NY, USA

**Keywords:** antifungals, amphotericin B, echinocandins, caspofungin, voriconazole, toll-like receptors

## Abstract

Fungi are increasingly recognized as major pathogens in immunocompromised individuals. With the increase in the number of fungal infections each year and the development of resistance to current therapy, new approaches to treatment including stimulation of the immune response in addition to concurrent pharmacotherapy is ongoing. The most common invasive fungal infections are caused by *Candida* spp., *Aspergillus *spp., and *Cryptococcus *spp. Amphotericin B (AmB) has remained the cornerstone of therapy against many fulminant fungal infections but its use is limited by its multitude of side effects. Echinocandins are a newer class of antifungal drugs with activity against *Candida* spp. and *Aspergillus* spp. and constitutes an alternative to AmB due to superior patient tolerability and fewer side effects. Due to their oral delivery, azoles continue to be heavily used for simple and complex diseases, such as fluconazole for candidal vaginitis and voriconazole for aspergillosis. The objective of this paper is to present current knowledge regarding the multiple interactions between the broad spectrum antifungals and the innate immune response, primarily focusing on the toll-like receptors.

## INTRODUCTION

Fungal species are ubiquitous in the environment an estimated 1.5 million are known to exist (Hube, 2009). Only a few species are actually true pathogens in humans. Opportunistic fungi can cause life threatening infections ranging from superficial to deep seated infections in immunocompromised patients. In developing countries fungal infections affect both immunocompromised and immunocompetent individuals in areas that are endemic to mycoses (Brown et al., 2012). Fungal infections have increased over the last few decades and this can be correlated to increased invasive medical management, immunosuppressed patients either from acquired infections or from treatment induced deficiencies (Pfaller and Diekema, 2007). Interestingly, fungi are the fourth main cause of hospital acquired infections in populations “at-risk” despite the availability of antifungal treatment. This point illustrates the need for further study to identify more efficient ways to combat these interesting pathogens. It is often challenging to treat fungal infections because current methods to identify particular species are not always reliable or accurate resulting in delayed or inappropriate treatment (Perlin, 2011; Pfaller, 2012).

Amphotericin B (AmB) is a polyene antifungal agent first isolated from *Streptomyces nodosus* in 1955, from Venezuelan soil samples near the Orinoco River region (Dutcher, 1968). AmB is selectively toxic toward fungal cells, displaying high affinity for ergosterol, subsequently destabilizing fungal membranes (refer to **Table 1** – “Commonly used systemic antifungal drugs” for mechanism of action of common antifungals). It is primarily used in systemic fungal infections caused by *Histoplasma, Coccidioides, Candida, Blastomyces, Rhodotorula, Cryptococcus, Sporothrix, Mucor and Aspergillus spp. *and the drug has remained the cornerstone of the therapy against fulminant fungal infections (Ellis, 2002). AmB also has activity against some protozoans, and prions (Adjou et al., 1997; Kafetzis et al., 2005). AmB is amphoteric as well as amphipathic, has a low therapeutic index, and is associated with significant dose-related nephrotoxicity (Fanos and Cataldi, 2000), as well as acute, infusion-related febrile reactions (Khoo et al., 1994). Lipid-based formulations of AmB have allowed patients to receive higher doses while sparing toxicity (Hiemenz and Walsh, 1996). AmB also stimulates the production of inflammatory cytokines (TNF-α, IL-1β, IL-6, IL-8), chemokines (MCP-1, MIP-1β, IL-8), prostaglandins, and nitric oxide (Cleary et al., 1992; Arning et al., 1995; Razonable et al., 2005). In addition to its direct antifungal activity, AmB activates toll-like receptors (TLRs), which contribute to the cytokine responses.

**Table 1:** Commonly used systemic antifungal drugs [as reviewed by Lewis (2011)].

Mechanism	Class	Drugs
**Cell membrane**	Azoles (14-α demethylaseinhibitors)	**Triazoles**
Ergosterol inhibitors/binders		Fluconazole, itraconazole, voriconazole, posaconazole,
	Polyenes (ergosterol binding)	Amphotericin B
	Allylamines (squalene monooxygenase)	Terbinafine
**Cell wall**	Echinocandins (β-1,3 D-glucan synthesis inhibitors)	Anidulafungin, caspofungin, micafungin
B-1,3 D-glucan synthesis		
**Intracellular**	Pyrimidine analogs/thymidylate synthase inhibitor	Flucytosine
	Mitotic inhibitor	Griseofulvin

Echinocandins are a newer class of antifungal drugs that display a unique mechanism of action, inhibiting the synthesis of 1,3-β-D-glucan in the cell wall, through the inhibition of the enzyme 1,3-β glucan synthase (Morris and Villmann, 2006; Fera et al., 2009). Besides having a structural role, β-D-glucan also demonstrates potent immunostimulatory properties mediated by the innate immune receptor Dectin-1, as well as TLRs and C-type lectin receptors, which are expressed on host cells (Brown, 2006; Wheeler et al., 2008). Following binding, echinocandins induce the activation of phagocytic and proinflammatory responses (Dennehy and Brown, 2007). The echinocandins’ antimicrobial spectrum includes most of the *Candida* spp. strains, *Aspergillus spp.*, and has some activity against *Pneumocystis jiroveci *(Denning, 2002). The advantages of echinocandins include long half-life allowing daily dosing, no dose adjustment in renal impairment or hemodialysis, minimal adverse effects, and limited drug interactions (Denning, 2002).

Human TLRs are closely related to the toll receptors in *Drosophila melanogaster*, and they are important for defense against microbial infection (Medzhitov et al., 1997). TLRs are a class of proteins that play a critical role in the immune systems response to invading pathogens. They are found in tissues involved in immune function, as well as in tissues exposed to the external environment (the respiratory and the gastrointestinal tract). Ten TLRs have been identified in humans (TLR1–TLR10). They recognize structural repeating sequences known as pathogen-associated microbial patterns which are expressed by microbial pathogens, or danger-associated molecular patterns that are endogenous molecules released from necrotic cells and stimulate the release of inflammatory cytokines (Newton and Dixit, 2012). Some of the most important TLRs are TLR1 which recognize pathogen-associated molecular pattern with a specificity for Gram-positive bacteria, TLR2s that recognize many bacterial, fungal, viral, and certain endogenous substances, TLR4 which detects lipopolysaccharide from Gram-negative bacteria and TLR9, which is expressed by numerous cells of the immune system such as dendritic cells, B lymphocytes, monocytes, and natural killer cells and recognizes unmethylated CpG sequences in DNA molecules.

## AMPHOTERICIN B AND TLRs

AmB stimulates multiple TLRs, namely TLR1, TLR2, TLR4. Sau et al. (2003) demonstrated that TLR2 and CD14 receptors play an important role in the release of the inflammatory cytokines TNF-α and IL-8. Moreover, TLR2 has a key role in the release of IL-1β. Peritoneal macrophages, isolated from murine cells lacking TLR2, failed to release TNF-α, IL-1β, and IL-8 in response to AmB stimulation, in comparison with peritoneal macrophages isolated from TLR2 positive mice, which displayed increased inflammatory cytokine production. Furthermore AmB induced TNF-α production was suppressed in peritoneal macrophages that expressed mutant, non-functional TLR4. However, this effect was observed only at higher AmB concentrations. The authors of the study also demonstrated that TLR response to AmB was CD14 dependent. Therefore, CD14 positive cells produced TNF-α when stimulated by AmB, whereas those which were CD14 negative did not. Interestingly, lipid formulations of AmB did not elicit significant cytokine production and release from murine peritoneal macrophages, possibly due to the low concentration of unbound, non-lipid associated AmB (Sau et al., 2003). 

The essential role of TLR1 in AmB induced cell activation was proven by Razonable et al. (2005) using THP1 monocytic cell line. The preincubation of THP1 cells with murine anti-human TLR1 monoclonal antibody (anti-TLR1 MAb) reduced the production of IL-6, IL-8, and TNF-α in response to AmB. Anti-TLR1 MAb also inhibited IL-8 secretion in response to the TLR2–TLR1 ligand Pam-3-*Cys*. Additionally, IL-8 inhibition with anti-TLR1 MAb was superior than with anti-TLR2 MAb and the addition of anti-TLR1 MAb augmented the degree of IL-8 inhibition by anti-TLR2 MAb (Razonable et al., 2005).

To further characterize the influence of AmB on TLRs, Bellocchio et al. (2005) showed that the expression of TLR2 and TLR4 was activated upon exposure of neutrophils (human and mice) to *Aspergillus* conidia. However, TLR4 was only stimulated upon exposure to the fungal hyphae. AmB increased the expression of TLR2, while liposomal AmB increased the expression of TLR4 in neutrophils. Using purified murine neutrophils, the authors were able to demonstrate that both TLR4 activation and liposomal AmB deter production of pro-inflammatory cytokines and stimulate anti-inflammatory cytokines. Additionally, in the absence of TLR4, liposomal AmB acts like deoxycholate AmB, stimulating the release of inflammatory cytokines (Bellocchio et al., 2005).

In another study published by Matsuo et al. (2006) using**monocyte-like cell lines, the authors demonstrated that AmB phosphorylates p65 of nuclear factor-kappaB. Further evidence in the study suggested that this leads to stimulation of proinflammatory cytokine production, mediated by receptors including TLR2 and NF-kappaB (Matsuo et al., 2006).

## ECHINOCANDINS AND TLRs

The influence of echinocandins on the innate immune receptors is achieved through the influence of these antifungals on β-D-glucan. A report published by Moretti et al. (2012) demonstrated that caspofungin influenced TLR2/Dectin-1 interactions, as wells as Dectin-1 engagement with TLR4 and TLR9. Using an invasive aspergillosis model in which two different strains of Dectin-1/TLR2 deficient murines were treated with caspofungin, the authors found that at lower concentration of the drug (0.1 mg/kg) the restricting activity on fungal growth was preserved, as well as the inflammatory cell recruitment. However, this was dependent on the genetic background of the host (C57BL/6 responded to treatment with caspofungin but BALB/c did not). At higher doses (5 mg/kg) both types of mutant mice had significant restriction of fungal growth and reduction of inflammatory cell recruitment. Both the protective (at 0.1 mg/kg) and the exacerbating (at 5 mg/kg) effects of caspofungin were lost in TLR2 deficient mice, indicating that TLR2 is required for the antifungal activity of echinocandins against aspergillosis. Furthermore, using TLR4 and TLR9 deficient mice with invasive aspergillosis, the authors showed that TLR4 contributes to the protective effect and TLR9 contributes to the exacerbating effect of caspofungin (Moretti et al., 2012).

Similarly, using a murine model of infective aspergillosis, Moretti et al. (2013) studied the immunomodulatory activity of echinocandins. Micafungin controlled cytokine response to *A. fumigatus* by decreasing the expression of TNF-α and increasing IL-10 release. The anti-inflammatory activity of micafungin required IL-10 and occurred through signaling via the TLR2/dectin-1 and TLR3/TRIF pathways (Moretti et al., 2013).

In another article published by Salvenmoser et al. (2010), caspofungin treatment resulted in the highest upregulation of TLR2 by *A. fumigatus *whereas exposure of *C. albicans* to caspofungin let to the significant upregulation of TLR4 and TLR9.

## AZOLES AND TLRs

There seems to be a similar interaction of azoles on TLR and subsequent immunomodulation, however, the data is limited. In previously mentioned article published by Salvenmoser et al. (2010), voriconazole treatment upregulated TLR2, TLR4, and TLR9 by *A. fumigatus*. In another study by Simitsopoulou et al. (2008) an additive antifungal effect was demonstrated when voriconazole was combined with monocytes and *A. fumigatus* hyphae. Both *A. fumigatus* hyphae and voriconazole induced increased expression of TLR2 as well as TNF-α in monocytic cells compared to untreated cells. The effects were seen when both were used independently but more significantly when used in combination. In contrast, TLR4 expression was not increased by either voriconazole or fungal hyphae. In addition, significantly more NF-kappaB was translocated to monocyte cell nuclei treated with voriconazole than untreated cells. The study suggests that TLR2 signaling, TNF-α, and NF-kappaB activation in the presence of voriconazole proposes an immunomodulation effect leading to a more efficient response to *A. fumigatus* (Simitsopoulou et al., 2008).

## DISCUSSION

Fungi are increasingly recognized as major pathogens in immunocompromised individuals. Risk factors for invasive fungal infections include prolonged neutropenia, hematological malignancy, transplantation (particularly bone marrow transplant), cytotoxic drugs, and steroid therapy (Enoch et al., 2006).

The most common invasive fungal infections are candidiasis, followed by aspergillosis and *cryptococcosis* (Shoham and Marr, 2012). Disseminated candidiasis is associated with a mortality in excess of 25% (Kibbler et al., 2003), and represents the fourth most common cause of nosocomial blood stream infection in United States (Wenzel and Edmond, 2001). Invasive aspergillosis is also associated with significant morbidity and mortality, lung, and heart–lung transplant recipients being at greatest risk of infection, affecting 14–18% of patients (Hagerty et al., 2003). AmB remains the most effective drug against fulminant fungal infections but its use is limited by the multitude of side effects including nephrotoxicity, infusion related toxicity, electrolyte abnormalities, and others. Echinocandins are a newer class of antifungal drugs with activity against *Candida* spp. and *Aspergillus* spp. and constitutes an alternative to AmB due to a superior toleration profile and less side effects. The azoles used for systemic fungal infections are triazoles and include fluconazole, itraconazole, voriconazole, and posaconazole. They inhibit the cytochrome P450 dependent enzyme lanosterol 14-alpha-demethylase, thus inhibiting the synthesis of ergosterol, which represents a vital component of the cellular membrane of fungi (Zonios and Bennett, 2008). As outlined in this review, AmB, echinocandins, and some of the studied azoles have long been used and their mechanisms of action against fungi are well established. However, these drugs also act on components of the innate immune system aiding in the body’s natural defense against the infecting pathogens. As outlined in this review, TLRs seem to be significant components in this setting. The innate immune response has physical barriers that provide protection from the environment which include the skin and mucus membranes of the respiratory, gastrointestinal, and genito-urinary tracts. Once fungi have invaded these barriers they encounter a multitude of innate defenses that include phagocytes, natural killer cells, T cells, B cells, and endothelial cells (Blanco and Garcia, 2008). Generally the interaction between antifungal therapy and the immune system is synergistic. Chemotactic factors are produced at the site of fungal infections, leading to activation of the complement pathway. The synthesis of these chemotactic factors is stimulated by pathogen associated molecular patterns, (PAMPs) that are recognized by pattern recognition receptors (PRRs). PRRs include TLRs. PRRs and TLRs activate PAMPs which signal the synthesis and release of pro-inflammatory cytokines that activate the adaptive immunity. A fungal pathogen activates multiple PRRs, which alerts the immune system to respond with a broad range of possibilities (Janeway and Medzhitov, 2002; Roeder et al., 2004; Romani,2004). Antimicrobial peptides are other components of the innate immune system that have an antimicrobial effect against fungi. There exact mechanism is not known but they are likely to activate and mediate the innate and adaptive immune response in infection and inflammation. They also inactivate fungi by directly affecting their membrane (Ganz, 2003;Aerts et al., 2008,Steinstraesser et al., 2008).

Polymorphisms in TLR genes have been associated with a susceptibility to fungal infections. The type of infection depends on which TLR has mutated. Recognition of *Candida* normally occurs through PRRs such as TLRs. Plantinga et al. (2012) analyzed that TLR single nucleotide polymorphisms (SNPs) [R80T, S248N, 1602S] on TLR1 were associated with candidemia in white populations. This was not present in African American populations but this was attributed to the lower power in the smaller study population. These polymorphisms also impaired cytokine release by monocytes (Plantinga et al., 2012).

Invasive aspergillosis is a particular concern in patients that have had hematopoietic-cell transplants, with its incidence rate increasing. Despite the availability of new medications (azoles and echinocandins) their outcome remains poor (Marr et al., 2002). Aspergillosis activates the immune system through TLR4. Bochud et al. (2008) analyzed that donor TLR4 haplotypes (S3, S4) increased the risk of invasive aspergillosis among recipients of allogenic hematopoietic-cell transplants.

The ability of antifungals to simultaneously elicit an efficient immune response should be researched further for the potential development of new drugs to induce effective activation of the innate immune system via TLRs and subsequent pathways that may synergistically help clear fungal infections.

## Conflict of Interest Statement

The authors declare that the research was conducted in the absence of any commercial or financial relationships that could be construed as a potential conflict of interest.
